# Comparative Clinical Effectiveness and Cost-Effectiveness of the Cochlear Osia System and Baha Attract System in Patients with Conductive or Mixed Hearing Loss or Single-Sided Deafness

**DOI:** 10.3390/jmahp12010003

**Published:** 2024-03-06

**Authors:** Matthias Brunner, Manjula Schou, Robert J. Briggs, Dell Kingsford Smith

**Affiliations:** 1 Cochlear Limited, Macquarie University, Sydney, NSW 2109, Australia; mbrunner@cochlear.com (M.B.);; 2 Departments of Surgery and Otolaryngology, University of Melbourne, Parkville, VIC 3052, Australia

**Keywords:** comparative, clinical effectiveness, cost-effectiveness, Osia System, hearing loss

## Abstract

The aim of this study was to evaluate the comparative clinical effectiveness and cost-utility of the active transcutaneous Osia^®^ System versus the passive transcutaneous Baha^®^ Attract System for patients with conductive or mixed hearing loss or single-sided deafness in an Australian healthcare setting. In the absence of direct comparative evidence, an indirect treatment comparison (ITC) of the clinical effectiveness and utility gains was needed. The ITC was informed by three studies identified through a systematic literature review. A Markov model was developed to evaluate the cost-utility of the Osia System. The literature review identified three studies suitable to inform an ITC: Mylanus et al. 2020 and Briggs et al. 2022 (Osia System) and den Besten et al. 2019 (Baha Attract System). The Osia System was found to be clinically superior to the Baha Attract System, across objective audiological outcomes resulting in a clinically meaningful utility benefit of 0.03 measured by the Health Utility Index with at least equivalent safety. In conclusion, the Osia System is more effective than the Baha Attract System, providing better hearing and health-related quality of life outcomes. In an Australian healthcare setting, the Osia System is cost-effective as demonstrated in a cost-utility analysis versus the Baha Attract System.

## 1. Introduction

Bone conduction hearing implants (BCHIs) have been available for four decades and are an established means of aural habilitation/rehabilitation for individuals with conductive hearing loss (CHL), mixed hearing loss (MHL) or single-sided deafness (SSD) [1,2] where conventional hearing aids can no longer provide a benefit or are contraindicated [1,3]. The original percutaneous and more recent passive and active transcutaneous systems are commercially available in Australia, with the choice of system dependent on the severity of hearing loss and user preference. Given the majority of the Australian population lives in a tropical or subtropical climate with an associated increased risk of peri-abutment infections [4,5], transcutaneous BCHIs have become the standard of care in Australia.

Active transcutaneous BCHIs have been designed to provide improved hearing outcomes while maintaining the aesthetic benefits and reduced risk of implant site infections of passive transcutaneous solutions for patients with CHL, MHL or SSD [1]. Unlike passive transcutaneous systems, the transducer used in active transcutaneous systems is implanted under the skin rather than residing within the external sound processor (SP), thus eliminating the potential for soft tissue attenuation which can significantly reduce sound transfer, particularly at the higher frequencies important for speech understanding [6]. However, unlike passive systems, active transcutaneous systems using an electromagnetic transducer typically require recessing the transducer into the skull bone [7], which may limit the optimal placement of the implant, require pre-operative computed tomography (CT) during surgical planning and may result in minor adverse events (AE) such as pain and skin infections [3,8]. A newer active transcutaneous BCHI, the osseointegrated steady-state implant system, with a fitting range of up to 55 dB hearing level (Cochlear Osia System, Cochlear Ltd., Sydney, Australia), employs a piezoelectric transducer instead of an electromagnetic transducer. Piezoelectric technology results in a thinner transducer that does not need to be recessed and can be fixed to the bone surface via established osseointegrated implant technology, thus reducing surgical complexity [1].

In Australia, a health economic evaluation is a mandated requirement for reimbursement approval for health insurance coverage via the Prostheses List. However, there is little economic evidence in the published literature on BCHIs and the cost-effectiveness of implantable devices. To this end, a cost-utility assessment of the Osia System was conducted to address the Australian funding requirements which request a new device to be “compared to alternative products on the Prostheses List or alternative treatments and:

(*i*) assessed as being, at least, non-inferior in terms of clinical effectiveness; and
(*ii*) the cost of the product is relative to its clinical effectiveness” [9].

Such an economic evaluation needs to be informed by sound clinical evidence. To date, there have been no large randomised controlled trials (RCTs) comparing different BCHI systems for disabling hearing loss that have a sufficient duration of patient follow-up and collected utility measures required to inform a cost-utility assessment. A small prospective randomised study with four patients assigned to each intervention, indicated that the Osia System was a better solution than the Baha Attract System for early audiological and quality of life outcomes [10]. The Gawecki et al. [10] study also showed a significant improvement in speech understanding with the Osia System compared with a non-implanted transcutaneous bone conduction device, Baha 5 Power on a Softband. However, the authors noted this study had limitations relating to the small number of patients, short duration (3 months) and baseline differences between groups in age and preoperative hearing status before implantation. 

The Osia System has been studied using a standard methodology in disabling hearing loss where patients serve as their own controls in two large, well-conducted, multicentre prospective clinical studies which included patients with CHL, MHL and SSD [1,2]. These studies concluded the Osia System is more effective than both the unaided hearing situation (for audiological outcomes and patient quality of life, including a utility measure Health Utilities Index Mark 3 (HUI3)) and a non-implanted transcutaneous Baha bone conduction system (for audiological outcomes only).

In the absence of direct comparative data in patients with CHL, MHL and SSD with a sufficient duration of follow-up and with utility measures to inform a cost-utility analysis, the aim of this study was to:

- conduct a systematic literature review to inform a robust indirect treatment comparison (ITC) of the clinical effectiveness of the Osia System with the Australian comparator BCHI, the passive transcutaneous Baha Attract System and
- construct a Markov cost-utility model to inform the cost-effectiveness of the Osia System versus the Baha Attract System, the transcutaneous BCHI most likely replaced in an Australian healthcare setting.

## 2. Materials and Methods 

### 2.1. Systematic Literature Review

#### 2.1.1. Study Eligibility Criteria

Electronic databases (Medline and Embase) were searched to identify systematic reviews, meta-analyses and clinical studies of the Osia System and the Baha Attract System published as of 6 October 2022. The search string used was (‘bone anchored hearing aid’ OR baha):de,ti,ab,dn OR (bone conduction OR bone anchored):ti,ab AND implant* OR fixture OR osseointegration OR abutment of prescription OR fitting OR ‘hearing aid*’ OR speaker OR ‘sound processor’):ti,ab,kw OR (‘fitting software’ OR apps OR wireless OR softband OR headband OR testband OR ‘hearing aid’/exp OR implant/exp or osseointegration/exp OR ‘osseointegrated implant’/exp).

All the papers were assessed against the participants, intervention, comparison, outcomes and study design (PICOS) framework by two independent authors with discrepancies resolved by discussion or consulting a third reviewer (Table 1). The inclusion criteria were:

(1) a comparative clinical study and
(2) a prospective arm of the Osia System and/or the Baha Attract System in the study and
(3) sample size was representative, i.e., ≥20 patients with CHL, MHL and/or SSD for Osia System and Baha Attract System and
(4) adequate information for review in English.

#### 2.1.2. Data Extraction

Data were extracted from the text or tables, or if data could be accurately calculated, from graphs or figures. Where required and available, the patient-level study data were accessed directly to overcome reporting asymmetry between the publications of the Osia System and the Baha Attract System.

### 2.2. Meta-Analysis of Study Endpoints and Indirect Treatment Comparison

#### Audiological Outcomes

In many BCHI studies results are reported separately for the group with a device placed on the hearing-affected side (CHL and MHL) and the group with a device placed on the contralateral side (SSD). Where outcomes were reported separately by type of hearing loss (CHL/MHL and SSD) and not for the overall cohort, first principles were employed to determine the mean and the standard deviation (SD) of the whole cohort using the summaries provided for the subgroups as follows:

$$\bar{Z}=\frac{m\bar{X}+n\bar{Y}}{m+n}$$

$$SD(Z)=\sqrt{\left[\frac{\left(m-1\right)VAR\left(X\right)+\left(n-1\right)VAR\left(Y\right)+m{\left(\bar{X}-\bar{Z}\right)}^{2}+n{\left(\bar{Y}-\bar{Z}\right)}^{2}}{m+n-1}\right]}$$

Here, *m* is the number of observations in the first subgroup (for instance the CHL/MHL subgroup), $\bar{X}$ is the mean outcome in the subgroup and *VAR*(*X*) is the variance of the outcome in the subgroup. Likewise, for the second subgroup (for instance the SSD subgroup), *n* is the number of observations in the subgroup, $\bar{Y}$ is the mean outcome in the subgroup and *VAR*(*Y*) is the variance of the outcome in the subgroup. From these, the mean ($\bar{Z}$) and standard deviation (*SD*[*Z*]) of the overall cohort can be determined from first principles.

A random effects inverse-variance weighted meta-analysis was utilised to synthesise the evidence generated from two or more studies. Finally, the mean difference in outcomes between the Osia System and the Baha Attract System was calculated as follows, where SE denotes the standard error:

$$Mean\;treatment\;difference\;\left(\bar{D}\right)=Mean\;change\;Osia\;System\;\left(\bar{O}\right)-Mean\;change\;Baha\;Attract\;System\;(\bar{B})$$

$$SE(\bar{D})=\sqrt{VAR\left(\bar{O}\right)+VAR(\bar{B})}$$

Statistical significance is evaluated at the 5% level, and no corrections for multiplicity of testing are made. 

### 2.3. Economic Evaluation

#### 2.3.1. Model Perspective and Structure

**Figure 1:**
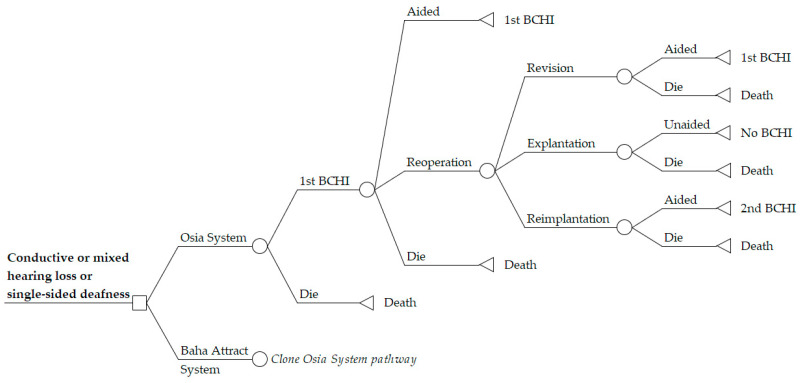
Markov model structure for cost-utility analysis of Osia System versus Baha Attract System. The model structure is equal in both model arms, depicted by “Clone Osia System Pathway” in the Baha Attract System arm. BCHI = bone conduction hearing implant.

A Markov economic model was developed in Microsoft Excel 2016 to estimate the cost-utility of the Osia System compared to the Baha Attract System in an Australian healthcare setting. The analysis was conducted from a healthcare perspective, only considering direct costs; costs borne outside the healthcare system such as time off work or travel to and from clinic visits were excluded. Costs and outcomes were discounted at 5% annually which is the standard rate for Australian economic evaluations [11,12]. Markov iterations were computed in quarterly cycles with half-cycle correction. Model pathways are equal for both arms with BCHI-specific transition probabilities at each node. Post initial implantation, patients remain in a first BCHI health state where they accrue costs and benefits according to aided hearing with their BCHI. Each cycle, patients are at risk of requiring reoperation to manage a serious device-related adverse event (SAE). Patients may undergo either device revision surgery, device explantation, or implantation of a replacement device. Post-reoperation, patients thus remain either aided with their revised first BCHI or a replacement second BCHI or transition to an unaided health state with no further hearing benefits from a BCHI. Age-matched background mortality derived from Australian life tables [13] was applied in all health states. The structure of the Markov model is depicted in Figure 1.

#### 2.3.2. Time Horizon

Costs and consequences were evaluated in the base case over a 10-year time horizon, reflecting the manufacturer warranty period for the Osia OSI200 implant. The model start age was set to the average age of the Osia System clinical study populations informing the model. 

#### 2.3.3. Choice of Health Outcomes

The HUI3 utilities associated with the two interventions were used to calculate Quality Adjusted Life Year (QALY) gains with the Osia System compared to the Baha Attract System. Patients with a hearing disability of a severity indicated for a BCHI have significant long-term hearing loss which is essentially permanent. Although some patients with this level of hearing loss may worsen slowly over 5–10 years [14], high-power BCHI systems continue to benefit patients for many years given that the system power levels do not degrade over time and can routinely be adjusted by an audiologist to maximise and maintain good hearing outcomes. Hence, utilities observed at least 6 months post-surgery were assumed to remain constant for the remainder of the model. Patients undergoing explantation of a BCHI reverted to the pre-operative, unaided hearing utility. Patients undergoing reimplantation entered a one-year tunnel state with time-dependent utilities reflecting the same rehabilitation phase as that following the initial procedure. The incremental cost-effectiveness ratio (ICER) was calculated by dividing the incremental costs of the Osia System versus the Baha Attract System by the incremental QALYs.

#### 2.3.4. Transition Probabilities and Adverse Event Rates

Model transition probabilities for reoperations (revision surgeries, explantations and reimplantations) and event probabilities for adverse events were derived from the clinical studies identified in the systematic literature search. Probabilities were assumed constant over the model time horizon. 

#### 2.3.5. Cost Inputs

Cost inputs included costs of the implant system surgery, anaesthesia, hospital stay, rate and cost of reoperations (revision, explantation or reimplantation) and AEs (soft tissue complications). The cost of SP upgrades was included at five-year intervals consistent with Australian clinical practice. All cost inputs were sourced from the latest available Australian tariffs, representative of the current costs in 2022, and are summarised in Table 2.

#### 2.3.6. Parameter Analysis

Univariate sensitivity analyses were performed to test the model for parameter uncertainty. Changes were made to the following parameters: time horizon (5 years and lifetime), discounting (2.5% and 7.5% for costs and outcomes), 10% higher or lower cost of procedures (surgery and hospitalisation) and management cost of AEs. In addition, the assumption of constant risk for reoperations and AEs was assessed by converging transition probabilities over a period of 12 months starting one year post-operatively, i.e., after two years the risk for reoperations or AEs was assumed to be equal in both model arms. Lastly, a sensitivity analysis was performed using the utility gains observed in CHL/MHL patients only, as these represent a majority of patients.

#### 2.3.7. Probabilistic Sensitivity Analysis

Probabilistic sensitivity analysis (PSA) was performed by Monte Carlo simulation (1000 model iterations) varying all model inputs using beta (baseline utilities and relative proportions of reoperation types), gamma (costs) and normal (demographic and utility gains) distributions.

## 3. Results

### 3.1. Search Results and Study Selection

**Figure 2:**
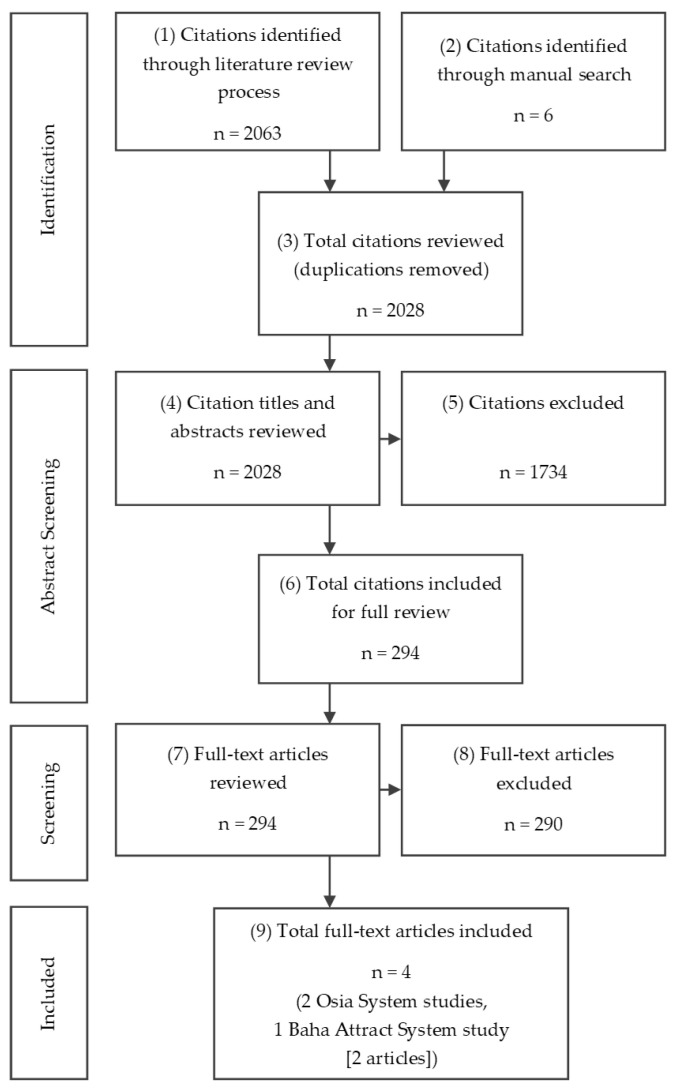
PRISMA chart of systematic literature review conducted.

The systematic search of electronic databases resulted in a total of 2063 articles. After screening, three studies were included, as shown in Figure 2.

#### Identified Studies

Two Osia System studies were identified, Mylanus et al. [1] and Briggs et al. [2]. One Baha Attract System study was published by den Besten et al. [18], with a longer-term extension of the den Besten et al. study reported by Kruyt et al. [19]. None of the three studies compared the Osia System directly with the Baha Attract System, although both these BCHI systems were compared with both unaided hearing and Baha SP technology on a Softband, a non-implantable bone conduction device that aids hearing (Table 3).

All three included studies consistently reported the following key health outcomes:

- objective audiological outcomes: threshold audiometry, speech performance in quiet and speech performance in noise,
- surgical and other AEs,
- patient-reported outcomes (PROs), and
- the HUI3, a measure considered to be most relevant to hearing.

All three included studies were single-arm studies with a before-and-after design where patients served as their own controls. That is, these studies did not contain a conventional concurrent control arm, either with no device (unaided) or using a non-implanted bone conduction device. The primary role of such a concurrent control arm would be to measure any changes or fluctuations in clinical outcomes (hearing and health-related quality of life (HRQoL)) that could occur naturally over time. However, when hearing loss is at a late stage in the clinical pathway where a BCHI is indicated, it is not likely that hearing loss will spontaneously improve. That is, unlike physiological ailments or disorders where natural changes and fluctuations do arise because of changes in the underlying biological mechanisms or through changes in lifestyle or interventions (e.g., exercise, change of diet, concomitant medications or similar), these changes are considered very unlikely to occur in hearing loss. This is, and has been, consistently recognised by regulatory authorities [21] who have over many years approved implantable hearing devices for effective and safe use in humans without the need for a concurrent control arm, but rather relied on a “before-and-after” implantation methodology with the “before” being a stable measure of hearing disability. Consequently, in this setting, an ITC using before-and-after studies is a reasonable method for comparison to inform the economic evaluation. 

As ITCs can be prone to bias, potential sources of bias need to be identified and addressed appropriately. To this end, the following are discussed in the sections that follow: study design level bias, patient level bias and study conduct level bias. 

Table 3 provides an overview of the study designs for the Osia System and Baha Attract System studies. The three studies are very similar in design with respect to the key inclusion criteria, the health systems across which they were conducted, the primary comparison of unaided hearing to measure response, and the range of audiological and quality of life endpoints, including the HUI3 measure and PROs. Thus, there is little difference between these studies at the design level. Consequently, it is anticipated that the likelihood of any systematic bias being introduced by the design of the studies is minimal. 

There were three audiological endpoints defined across all three studies: pure tone average (0.5, 1, 2 and 4 kHz) (PTA4), speech discrimination in quiet and speech discrimination in noise. It is evident from the summary presented in Table 3 that PTA4 and speech discrimination in quiet were conducted similarly across all three studies. However, with regards to speech discrimination in noise, the noise was presented differently; the noise was presented from behind in the Mylanus et al. [1] and den Besten et al. [18] studies whereas it was presented from the front in the Briggs et al. [2] study. This difference has the potential to introduce a systematic bias in the comparison between the Osia System and the Baha Attract System. For this reason, it was considered appropriate to not include the Briggs et al. [2] study in a comparison between the Osia System and the Baha Attract System for this endpoint. 

Table 4 summarises the key baseline characteristics of the patients enrolled in the three studies. A total of 80 patients were included for the Osia System and 54 for the Baha Attract System. Patient baseline characteristics resulting from the key inclusion criteria across the studies were comparable; similar average age, similar mean PTA4 levels and the majority of patients presented with CHL/MHL. This, therefore, provides further confidence that the likelihood of bias will be low in an ITC of the Osia System versus the Baha Attract System. 

In conclusion, the available evidence did not identify any sources of systematic bias of concern in the three studies, except for the impact of the presentation of the noise in the speech discrimination in noise measurement for the Briggs et al. [2] study. Thus, an ITC of the Osia System versus the Baha Attract System was undertaken.

### 3.2. Results of the Indirect Treatment Comparison

#### 3.2.1. Audiological Outcomes 

For PTA4, the Osia System consistently provided better hearing performance at 6 months, demonstrating a larger benefit than the Baha Attract System (28.07 dB compared with 21.02 dB) (Table 5). This PTA4 improvement was significantly in favour of the Osia System compared with the Baha Attract System (7.05 dB; 95% confidence interval (CI): [3.58 dB, 10.51 dB]).

In terms of speech discrimination in quiet, the Osia System consistently provided better hearing performance compared to the Baha Attract System at 6 months (Table 5). The change from baseline to 6 months for speech in quiet was 58.8% for the Osia System compared with 43.44% for the Baha Attract System and this improvement significantly favoured the Osia System (15.35%; 95% CI: [4.39%, 26.32%]).

Similar to the PTA4 and speech discrimination in quiet outcomes, the Osia System provided statistically significantly better hearing performance in terms of speech discrimination in noise at 6 months compared to the Baha Attract System (Table 5). The change from baseline to 6 months for speech in noise was higher for the Osia System (13.7 dB) compared with the Baha Attract System (4.26 dB) and this improvement was significantly in favour of the Osia System (9.44 dB; 95% CI: [6.5 dB, 12.39 dB]).

#### 3.2.2. Utilities 

The HUI3 is an accepted measure of HRQoL in hearing loss studies and there is evidence to demonstrate that the HUI3 is acceptable, reliable, and responsive to clinically meaningful change. An improvement of 0.03 or more in HUI3 utility represents a clinically meaningful change [22].

The HUI3 was collected as a secondary outcome in the three studies. HUI3 values collected 6 months post-intervention are considered to be reasonable to measure the impact of a BCHI. An Osia System study [2] and the den Besten et al. [18] Baha Attract System study reported HUI3 results at 6 months. The longer-term follow-up of the Baha Attract System study also collected HUI3 at 24 months [19]. The second Osia System study [1] reported results at 12 months. Due to the asymmetry in the collection time points of the HUI3, only the 6-month results are presented for the ITC.

Table 6 provides a summary of the utility changes measured when aided with the Osia System and Baha Attract System, compared to the unaided situation. Results are shown for all patients and separately for the CHL/MHL subgroup. At 6-month follow-up, HUI3 values showed a statistically significant improvement for both systems compared with the baseline unaided situation. However, the magnitude of the change in HUI3 values with the Osia System is greater than that for the Baha Attract System, with clinically meaningful numerical improvements with the Osia System versus the Baha Attract System at 6 months of 0.03 for all patients and 0.06 for the CHL/MHL group. The CHL/MHL subgroup represents the majority of the patients in this study cohort. 

#### 3.2.3. Patient Reported Outcomes

Patient-reported outcomes were collected as secondary outcomes in the three studies, with the Briggs et al. [2] Osia System study and the Baha Attract study reporting results at 6 months, and the Mylanus et al. [1] study reporting results at 12 months. A comparison was made with the pre-operative unaided setting.

The Abbreviated Profile of Hearing Aid Benefit (APHAB) is a self-reported assessment of hearing outcome. The APHAB questionnaire has 24 predetermined questions regarding various situations of aided hearing and is scored before and after fitting hearing devices. A summary of the APHAB scores is shown in Table 7, with the Osia System showing a 3-point greater numerical improvement versus Baha Attract, when both were compared to unaided hearing.

Speech, Spatial and Qualities of Hearing Scale (SSQ) is another self-reported assessment of outcome. The scores for the individual scales were reported in the three studies, the results are presented in Table 7. In all three SSQ scales the Osia System showed a greater numerical improvement than Baha Attract, when both were compared to unaided hearing.

#### 3.2.4. Adverse Events

A summary of the cumulative AEs relating to the device or procedure as reported in the three studies is shown in Table 8. 

A majority of AEs were classified as mild, including anticipated post-surgery pain and/or swelling which resolved within the study follow-up and without treatment. AEs of moderate severity occurred less frequently and, consistent with the BCHI system differences outlined earlier were mainly soft tissue complications which occurred at a higher rate with the Baha Attract System than with the Osia System. For the Baha Attract System, four events relating to magnet pressure/soft tissue problems were reported in four patients at 6 months. These soft tissue complications required local medical treatment but were resolved before the end of the follow-up period. 

Regarding SAEs for the Osia System, one patient in the Mylanus et al. [1] study experienced three SAEs, all of which were related to the implant procedure, rather than the device. The patient initially developed an infection at the implant site, and then an infection-related skin complication. This subsequently led to device explantation at 55 days post-surgery. In the Briggs et al. [2] study, no SAEs were reported up to 6 months after surgery, and no explantation or reimplantation procedures were reported. 

For the Baha Attract System in the den Besten et al. [18] study, one patient with a device-related AE had the implant removed due to infection at the implant site appearing shortly after implantation. No reimplantation procedures were conducted. At the 24-month follow-up for the Baha Attract System, a total of four serious AEs in four patients were reported [19]. All four patients subsequently had the implant surgically removed: two (approximately 4% of the total) due to persistent pain, one due to infection and one due to insufficient benefit. Two of these four subjects were later reimplanted with conversion to a percutaneous BCHI system.

Soft tissue complication rates at 6 months were calculated using the data in Table 8 for the economic model. In the Osia System studies, three events in 80 patients were reported over a total of 786 exposure months, resulting in a monthly complication rate of 0.38%. Four events observed with the Baha Attract System in 54 patients with 324 exposure months resulted in a monthly soft tissue complication rate of 1.23%. As discussed earlier, one subject in the Mylanus et al. [1] Osia System study experienced three SAEs, all of which were related to the implant procedure, rather than the device. No SAEs were reported in Briggs et al. [2] up to 6 months after surgery, and no reoperations were reported resulting in an overall monthly reoperation (explantation) rate for the Osia System of 0.13% (1 in 786 exposure months). At the 24-month follow-up with the Baha Attract System, 4 out of 54 patients underwent reoperation (1296 exposure months) [19]. Two patients had the implant surgically removed, one due to an SAE (infection at implant site) shortly after implantation and one due to persisting pain resulting in non-use. Two patients were converted to percutaneous systems, resulting in a total reoperation rate of 0.31% per month, of which one-half required explantation and the other half a reimplantation of their BCHI.

### 3.3. Base Case Results of the Economic Evaluation

The undiscounted and discounted deterministic results of the cost-utility model are presented in Table 9. Over a 10-year time horizon, the Osia System represented a cost-effective use of healthcare resources for patients with CHL, MHL and SSD. Compared to the Baha Attract System, the ICER for the Osia System was $29,301/QALY gained, well below the implicit Australian willingness to pay (WTP) threshold of $50,000/QALY gained [23].

### 3.4. Results of Sensitivity Analyses

#### 3.4.1. Parameter Analysis Results

**Figure 3:**
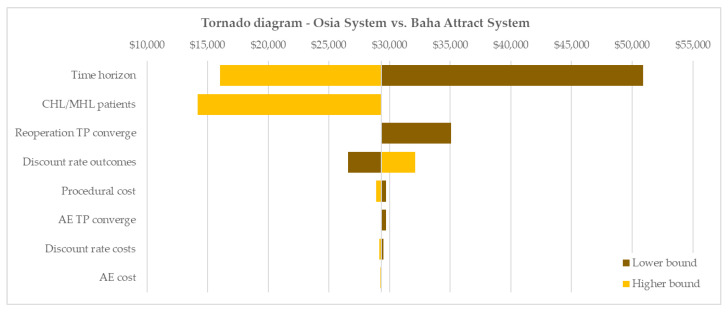
Tornado diagram of univariate sensitivity analyses. Base case ICER: $29,301/QALY gained. AE = adverse event, CHL = conductive hearing loss, MHL = mixed hearing loss, TP = transition probability.

The univariate parameter analyses demonstrated that the ICER was most sensitive to variation in the time horizon of the analysis. A short 5-year time horizon led to an increased ICER of $50,895. This still suggests borderline cost-effectiveness at the Australian WTP. In contrast, at a lifetime horizon, the ICER significantly reduced to $16,004 demonstrating increasing cost-effectiveness beyond the warranty-based base case time horizon of 10 years. The tornado diagram (Figure 3) further depicts the markedly reduced ICER achieved for the patient population with CHL or MHL ($14,161/QALY gained) due to increased incremental utility gains in this patient group compared with the small number of patients with SSD. Further univariate sensitivity analyses showed limited to negligible impact of variation in model inputs with ICERs remaining well below the Australian WTP of $50,000 per QALY gained [23].

#### 3.4.2. Probabilistic Sensitivity Analysis Results

**Figure 4:**
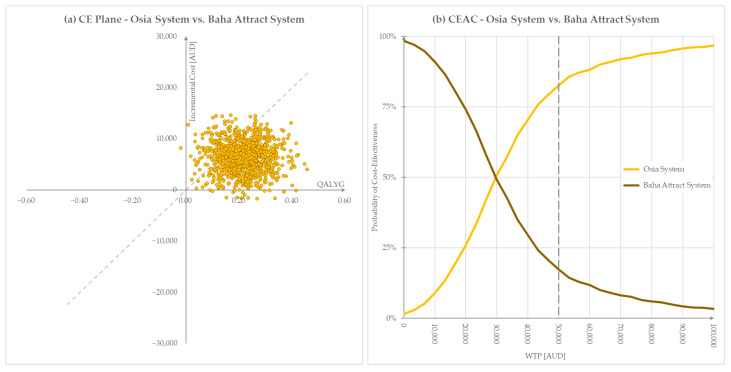
Graphical representation of PSA results, willingness to pay threshold $50,000. (**a**) Cost-effectiveness plane, (**b**) Cost-effectiveness acceptability curve. AUD = Australian dollars, QALYG = quality-adjusted life years gained, WTP = willingness to pay.

Probabilistic sensitivity analysis demonstrated that the model is robust and that the deterministic base case result was a reliable estimate of the cost-effectiveness of the Osia System versus the Baha Attract System. Monte Carlo simulations (1000 cycles) yielded distributions of incremental costs and QALYs over the 10-year model time horizon, shown in the cost-effectiveness plane in Figure 4a. The cost-effectiveness acceptability curve (CEAC) in Figure 4b demonstrates that at the Australian WTP of $50,000/QALY gained, more than 80% of all Monte Carlo iterations returned a cost-effective ICER.

## 4. Discussion

### 4.1. Clinical Effectiveness

Treatment with BCHIs plays an important role for patients with CHL or MHL or SSD where conventional hearing aids can no longer provide benefits or are contraindicated. Both the Osia System and Baha Attract System are indicated for these patients, and both have been shown to be significantly superior to unaided hearing for audiological and health-related quality of life outcomes [1,2,18,19].

In the absence of direct comparative clinical studies, we conducted a systematic review to identify studies that could be included in a robust indirect comparison of the Osia System and the Baha Attract System. This ITC confirmed that in objective audiological outcomes, the Osia System was consistently superior to the Australian comparator BCHI, the Baha Attract System, and demonstrated a clinically meaningful improvement in HRQoL as measured by the HUI3.

A broad literature search, using PICOS methodology to capture all relevant evidence sources followed by a systematic literature review that conformed to accepted standards was conducted [24]. While this systematic review did not identify adequate studies with direct evidence comparing the Osia System with the Baha Attract System, it identified three well-conducted, multicentre trials of the Osia and Baha Attract Systems. This necessitated an ITC of these systems. This ITC was conducted on the basis that an investigation of the potential sources of bias (study design level, patient level and study conduct level biases) did not identify any noteworthy differences, but indeed confirmed strong similarities between the studies. The ITC demonstrated that the Osia System consistently led to better hearing performances than the Baha Attract System at 6 months; significant improvements in PTA4, speech discrimination in quiet (65 dB) and speech discrimination in noise were observed. Additionally, these benefits were reflected in the patient’s overall quality of life with a utility increment of 0.03, a difference that is considered to be clinically meaningful, observed in favour of the Osia System at 6 months [22]. The improvements in hearing outcomes estimated by the ITC sit consistently with those observed at 3 months in a recently published study of eight subjects where subjects were randomised in a 1:1 ratio to the Osia System or the Baha Attract System [10]. Our ITC did not include the data from this study given the small sample size, short duration, and the lack of HRQoL data required to inform an economic evaluation.

### 4.2. Cost-Effectiveness

Health economic evaluation of interventions is increasingly required to inform clinician and payer decision-making in many global healthcare settings. However, there is little economic evidence in the published literature on BCHI, and to our knowledge, this is the first published analysis to evaluate the cost-effectiveness of the Osia System.

A review of the cost-effectiveness of BCHI conducted in 2016 concluded that bone-anchored hearing aids are cost-effective when considered for the appropriate indication [25]. More recently, a formal health technology assessment conducted by Ontario Health (Quality) in Canada concluded that for CHL or MHL, bone-conduction implants may be cost-effective compared with no hearing aids or no implant [26]. Another recent economic analysis of a transcutaneous BCHI has shown that, when compared to percutaneous BCHI, total accrued healthcare costs converged over the long term despite significantly higher upfront costs of the transcutaneous BCHI [27].

We conducted a robust cost-utility analysis for the Osia System utilising data systematically collected from large, prospective, multicentre clinical studies [1,2,18,19]. A strength of the analysis was that all studies identified in the systematic review were comparable clinical investigations that recruited similar patients, presented equivalent endpoints and included the same health utility measure. This allowed an ITC to be conducted. These data were used to inform an economic model to estimate the cost-utility of the Osia System compared to the Baha Attract System in an Australian healthcare setting. The model demonstrated that the Osia System is cost-effective compared to the Baha Attract System with an ICER of $29,301/QALY gained within the Australian healthcare system over a 10-year time horizon.

The limitations of our economic evaluation include uncertainty around the rate of implant device failures beyond the study observation periods and the rate of AEs; the base case model assumed a constant rate of AEs over the time horizon. However, sensitivity analyses around these parameters confirmed the cost-effectiveness of the Osia System across all scenarios. Importantly, only direct costs and savings related to the Australian healthcare system were considered in line with Australian health technology assessment standards. The exclusion of societal and indirect costs, such as medical-related travel costs, out-of-pocket payments, productivity change, educational costs, and disease burden may impact the analysis and should be considered in future economic evaluations of the Osia System for a more holistic assessment of its cost-effectiveness.

## 5. Conclusions

In the absence of adequate direct comparative studies, an ITC, utilising audiological and utility data from well-conducted, large clinical studies, demonstrated that the Osia System is more effective than the Baha Attract System, resulting in improved hearing outcomes for CHL, MHL and SSD patients, which translate into a meaningful improvement in HRQoL. The subsidy of the Osia System represents the cost-effective use of healthcare funds in Australia as demonstrated in a cost-utility analysis versus the treatment most likely to be replaced, the Baha Attract System.

## Figures and Tables

**Table 1:** Literature search PICOS.

Participants	CHL or MHL: pure tone average bone-conduction hearing threshold PTA4 ≤ 55 dB HL. SSD: pure tone average air-conduction hearing threshold of the contralateral ear PTA4 ≤ 20 dB HL.
Intervention	Osia System
Comparison	Baha Attract System at fitting range equivalent to the Osia System (up to 55 dB HL), as this is the transcutaneous BCHI most likely to be replaced by the Osia System in the Australian healthcare setting.
Outcomes	Audiological outcomes of pure tone audiometry, speech performance in quiet and speech performance in noise, compared with unaided hearing. The HUI3, considered to be most relevant to hearing disability, compared with unaided hearing. Surgical and other device and procedure-related AEs.
Study Design	Prospective arm of either the Osia System or Baha Attract System. Non-systematic reviews, studies with retrospective arms of either BCHI system, case reports, letters, editorials, animal/invitro studies and prospective clinical studies with n < 20 CHL and/or MHL and/or SSD patients were excluded.

AE = adverse event, BCHI=bone conduction hearing implant, CHL = conductive hearing loss, HL = hearing level, HUI3 = Health Utilities Index Mark 3, MHL = mixed hearing loss, PTA4 = pure tone average (0.5, 1, 2 and 4 kHz), SSD = single-sided deafness.

**Table 2:** Cost inputs for economic evaluation.

Cost Item (AUD)	Osia System	Baha Attract System	Source
BCHI system	$15,125	$8571	Prostheses List Benefit; Osia System (CO089); Baha Attract System: BI300 (CO051) + BIM400 (CO068) + Baha 6 Max (CO087) [15]
Replacement sound processor	$7166	$6484	Prostheses List Benefit; Osia System: Assumption (Benefit of SAMBA 2 Audio Processor (US029)); Baha: Baha 6 Max (CO087) [15]
Surgery	$1002	$1002	MBS; Items 41,603 (osseo-integration of fixture) + 41,604 (fixation of transcutaneous implant) + 20,120 (initiation of anaesthesia) + 23,085 (anaesthesia perfusion time 1:46–2:00 h) [16]
Hospitalisation	$5415	$5415	Approximation from public AR-DRG; D12B (Other Ear, Nose, Mouth and Throat Interventions, Minor Complexity, minus Prosthesis component) [17]
AE (soft tissue complication)	$138	$138	MBS; Items 104 (initial specialist visit) + 105 (subsequent specialist visit) [16]

AE = adverse event, AR-DRG = Australian Refined Diagnosis Related Groups, AUD = Australian dollars, BCHI = bone conduction hearing implant, MBS = Medicare Benefits Schedule.

**Table 3:** Included studies: Comparison of study designs.

Study Characteristics	Osia System		Baha Attract System
Reference	Mylanus et al. [1]	Briggs et al. [2]	den Besten et al. [18], Kruyt et al. [19]
Intervention	Osia System ^a^	Osia System ^a^	Baha Attract System
Primary comparator	Pre-operative unaided hearing condition		
Key inclusion criteria	Adults (18 years or older)		
	CHL or MHL with BC threshold of PTA4 ≤ 55 dB or SSD with AC threshold of PTA4 ≤20 dB in the contralateral ear		CHL or MHL with BC threshold of PTA4 < 30 dB or SSD with PTA4 ≤ 30 dB (≤ 20 dB for USA) in the contralateral ear
Study design	Open, single cohort, before-and-after, prospective, multicentre clinical investigation		
Countries	Australia, Germany, the Netherlands, Poland, USA	Australia, Hong Kong	The Netherlands, Poland, United Kingdom, USA
Reporting period	Primary: efficacy 3 months, safety 6 months; final: 12 months	Primary: efficacy and safety 3 months; final: 6 months	Primary: efficacy and safety 6 months; final: 24 months
Key clinical endpoints	Investigational device vs. unaided hearing (primary comparison) and Baha on Softband (secondary comparison for audiological outcomes)		
	Threshold audiometry, free field (PTA4, mean of 0.5, 1, 2 and 4 kHz)		
	Speech in quiet (50 dB, 65 dB and 80 dB SPL)		
	Adaptive speech recognition in noise with S0N180 (50% SNR)	Adaptive speech recognition in noise with S0N0 (50% SNR)	Adaptive speech recognition in noise with S0N180 (50% SNR)
	Self-reported assessments: Profile of Hearing Aid Benefit (APHAB), Health Utilities Index (HUI3), Speech, Spatial and Qualities of Hearing Scale (SSQ)		

^a^ In the Mylanus et al. study patients were implanted with the OSI100 Implant, in the Briggs et al. study patients were implanted with the commercial OSI200 Implant. OSI100 and OSI200 have been determined to be substantially equivalent by the FDA [20]. AC = air conduction, BC = bone conduction, CHL = conductive hearing loss, MHL = mixed hearing loss, PTA4 = pure tone average (0.5, 1, 2 and 4 kHz), SNR = signal-to-noise ratio, SPL = sound pressure level, SSD = single-sided deafness, S0N0 = speech and noise from front, S0N180 = speech from front, noise from behind.

**Table 4:** Comparison of key baseline patient characteristics.

Baseline Characteristics	Osia System		Baha Attract System
Reference	Mylanus et al. [1]	Briggs et al. [2]	den Besten et al. [18], Kruyt et al. [19]
Number enrolled	51	29	54
Age (years), mean (SD)	47.4 (14.7)	46.7 (19.7)	42.1 (13.6)
Gender, n (%)	Male: 27 (52.9%) Female: 24 (47.1%)	Male: 13 (44.8%) Female: 16 (55.2%)	Male: 21 (38.9%) Female: 33 (61.1%)
Type of hearing loss, n (%)	CHL/MHL: 37 (72.6%) SSD: 14 (27.5%)	CHL/MHL: 24 (82.8%) SSD: 5 (17.2%)	CHL/MHL: 39 (72.2%) SSD: 15 (27.8%)
Pre-operative PTA4 unaided hearing (dB), mean (SD)	53.9 (11.6) ^a^	53.6 (11.3) ^a^	51.9 (10.5) ^b^

^a^ Data extracted from patient level data sets, ^b^ Calculated using first principles from the summaries reported for the CHL/MHL and SSD subgroups. CHL = conductive hearing loss, MHL = mixed hearing loss, PTA4 = pure tone average (0.5, 1, 2 and 4 kHz), SD = standard deviation, SSD = single-sided deafness.

**Table 5:** Audiological outcomes at 6 months—Osia System versus Baha Attract System through unaided hearing.

**Audiological Outcomes** **Mean (SD), N; 95% CI**	**Osia System**			**Baha Attract System**	**Osia System versus Baha Attract System**
Reference	Mylanus et al. [1]	Briggs et al. [2]	Meta-analysis	den Besten et al. [18], Kruyt et al. [19]	
PTA4 (dB)					
Pre-operative	53.90 (11.60), 51 ^a^	53.60 (11.30), 29 ^a^	53.79; 51.27, 56.31	51.94 (10.46), 54; 49.15, 54.73 ^b^	
Change at 6 months	27.90 (9.10), 49 ^a^	28.40 (9.60), 28 ^a^	28.07; 26.00, 30.14	21.02 (10.41), 54; 18.25, 23.80 ^b^	7.05; 3.58, 10.51
Speech discrimination in quiet (65 dB) (%)					
Pre-operative	25.50 (25.40), 51 ^a^	37.80 (30.30), 29 ^a^	30.88; 18.92, 42.84	46.76 (32.62), 54; 38.06, 55.46 ^b^	
Change at 6 months	61.50 (27.70), 48 ^a^	54.00 (29.80), 28 ^a^	58.80; 51.74, 65.86	43.44 (31.47), 54; 35.05, 51.84 ^b^	15.35; 4.39, 26.32
Speech discrimination in noise (presented from behind) (dB)					
Pre-operative	4.98 (7.76), 51; 2.85, 7.11 ^a^	NR	NA	8.57 (6.26), 36; 6.52, 10.61 ^b^	
Change at 6 months	13.70 (8.10), 48; 11.41, 15.99 ^a^	NR	NA	4.26 (5.66), 36; 2.41, 6.11 ^b^	9.44 (6.5, 12.39)

^a^ Data extracted from patient level data sets, ^b^ Calculated using first principles from the summaries reported for the CHL/MHL and SSD subgroups. CI = confidence interval, NA = not applicable, NR = not reported, PTA4 = pure tone average (0.5, 1, 2 and 4 kHz), SD = standard deviation.

**Table 6:** Change in health utility index over time.

HUI3 Mean (SD), N; 95% CI	Osia System			Baha Attract System
Reference	Mylanus et al. [1]	Briggs et al. [2]	Meta-analysis	den Besten et al. [18], Kruyt et al. [19]
All patients				
Pre-operative	0.65 (0.22), 46; 0.59, 0.71 ^a^	0.69 (0.23), 29; 0.61, 0.77 ^a^	0.67; 0.61, 0.72	0.66 (0.24), 52; 0.60, 0.73
Change at 3 months	0.08 (0.23), 42; 0.01, 0.15 ^a^	0.10 (0.17), 27; 0.03, 0.16 ^a^	0.09; 0.04, 0.14	NR
Change at 6 months	NR	0.09 (0.17), 27; 0.03 ^a^, 0.15 ^a^	NA	0.06 (0.25), 47; −0.01, 0.13
CHL/MHL patients				
Pre-operative	0.61 (0.22), 34; 0.53, 0.68 ^a^	0.65 (0.22), 24; 0.56, 0.73 ^a^	0.62; 0.56, 0.68	0.67 (0.21), 37; 0.60, 0.74
Change at 3 months	0.10 (0.24), 30; 0.02, 0.19 ^a^	0.12 (0.17), 23; 0.05, 0.19 ^a^	0.11; 0.06, 0.16	NR
Change at 6 months	NR	0.12 (0.15), 23; 0.06, 0.19 ^a^	NA	0.06 (0.23), 34; −0.01, 0.14

^a^ Data extracted from patient-level data sets. CHL = conductive hearing loss, CI = confidence interval, HUI3 = Health Utilities Index Mark 3, MHL = mixed hearing loss, NA = not available, NR = not reported, SD = standard deviation.

**Table 7:** Change in patient-reported outcomes.

Mean Improvement vs. Pre-Operative (SD)	Osia System		Baha Attract System
Reference	Mylanus et al. [1] N = 48, reported at 12 months	Briggs et al. [2] N = 27, reported at 6 months	den Besten et al. [18] N = 53, reported at 6 months
Abbreviated Profile of Hearing Aid Benefit (APHAB)			
Global Scale	26.3 (18.5)	25.9 (26.2)	22.9 (18.1)
Speech, Spatial and Qualities of Hearing Scale (SSQ)			
SSQ Speech Scale	2.94 (1.94)	2.68 (1.89)	2.5 (1.7)
SSQ Spatial Scale	2.95 (2.52)	2.30 (2.42)	1.9 (1.9) (N = 52)
SSQ Qualities Scale	2.13 (2.30)	2.41 (1.81)	1.7 (1.4)

SD = standard deviation.

**Table 8:** Device- or procedure-related adverse events.

Adverse Events	Osia System		Baha Attract System
Reference	Mylanus et al. [1]	Briggs et al. [2]	den Besten et al. [18], Kruyt et al. [19]
Follow-up period	12 months	6 months	6 months, 24 months
Subjects, N	51	29	54
Soft tissue complications (classified as moderate severity AE)	1 event in 1 subject	2 events in 2 subjects	4 events in 4 subjects [11]
Reoperation/Serious AE	1 explantation due to 3 serious AEs	None	2 explantations (magnet removal, 1 due to serious AE) 2 reimplantations (conversion to percutaneous BCHI) [12]

AE = adverse event, BCHI = bone conduction hearing implant.

**Table 9:** Deterministic cost-utility model results.

Deterministic Results	Osia System	Baha Attract System	Incremental Change	ICER
Undiscounted				
Total costs (AUD)	$35,084	$28,630	$6453	$24,301 per QALY gained
Total QALYs	7.50	7.24	0.27	
Discounted				
Total costs (AUD)	$31,605	$25,257	$6348	$29,301 per QALY gained
Total QALYs	6.09	5.88	0.22	

AUD = Australian dollars, ICER = incremental cost-effectiveness ratio, QALY = quality adjusted life year.

## Data Availability

Where patient-level study data are used, these remain company confidential with data on file. All other data were extracted from published sources. Requests to access the datasets not already in the public domain should be directed to M.B.

## References

[1] Mylanus E.A.M., Hua H., Wigren S., Arndt S., Skarzynski P.H., Telian S.A., Briggs R.J.S. (2020). Multicenter Clinical Investigation of a New Active Osseointegrated Steady-State Implant System. Otol. Neurotol..

[2] Briggs R., Birman C.S., Baulderstone N., Lewis A.T., Ng I.H., Östblom A., Rousset A., Tari S., Tong M.C., Cowan R. (2022). Clinical Performance, Safety, and Patient-Reported Outcomes of an Active Osseointegrated Steady-State Implant System. Otol. Neurotol..

[3] Kirkby-Strachan G., Que-Hee C. (2016). Implantable hearing devices—An update. Aust. J. Gen. Pract..

[4] Ueda C.H.Y., Soares R.M., Jardim I., Bento R.F. (2022). Assessment Protocol for Candidates for Bone-Anchored Hearing Devices. Int. Arch. Otorhinolaryngol..

[5] Chan K.-C., Wallace C.G., Ho V.W.-Y., Wu C.-M., Chen H.-Y., Chen Z.-C. (2019). Simultaneous auricular reconstruction and transcutaneous bone conduction device implantation in patients with microtia. J. Formos. Med. Assoc..

[6] Motlagh Zadeh L., Silbert N.H., Sternasty K., Swanepoel W., Hunter L.L., Moore D.R. (2019). Extended high-frequency hearing enhances speech perception in noise. Proc. Natl. Acad. Sci. USA.

[7] Magele A., Schoerg P., Stanek B., Gradl B., Sprinzl G.M. (2019). Active transcutaneous bone conduction hearing implants: Systematic review and meta-analysis. PLoS ONE.

[8] Plontke S.K., Götze G., Wenzel C., Rahne T., Mlynski R. (2020). Implantation of a new active bone conduction hearing device with optimized geometry. HNO.

[9] Australian Government Department of Health and Aged Care (2020). Prosthesis List Guide 2020.

[10] Gawęcki W., Gibasiewicz R., Marszał J., Błaszczyk M., Gawłowska M., Wierzbicka M. (2022). The evaluation of a surgery and the short-term benefits of a new active bone conduction hearing implant—The Osia^®^. Braz. J. Otorhinolaryngol..

[11] PBAC (2016). Guidelines for Preparing a Submission to the Pharmaceutical Benefits Advisory Committee.

[12] MSAC (2021). Guidelines for Preparing Assessments for the Medical Services Advisory Committee.

[13] WHO (2019). Australian Life Tables.

[14] Wiley T.L., Chappell R., Carmichael L., Nondahl D.M., Cruickshanks K.J. (2008). Changes in hearing thresholds over 10 years in older adults. J. Am. Acad. Audiol..

[15] Australian Government Department of Health and Aged Care (2022). Prostheses List.

[16] Australian Government Department of Health and Aged Care (2022). Medicare Benefits Schedule.

[17] Independent Health and Aged Care Pricing Authority (IHACPA) (2020). National Hospital Cost Data Collection (NHCDC) Public Hospitals Report—Round 24 (Financial Year 2019–20) Appendix AR-DRG Version 10.0.

[18] den Besten C.A., Monksfield P., Bosman A., Skarzynski P.H., Green K., Runge C., Wigren S., Blechert J.I., Flynn M.C., Mylanus E.A.M. (2019). Audiological and clinical outcomes of a transcutaneous bone conduction hearing implant: Six-month results from a multicentre study. Clin. Otolaryngol..

[19] Kruyt I.J., Monksfield P., Skarzynski P.H., Green K., Runge C., Bosman A., Blechert J.I., Wigren S., Mylanus E.A.M., Hol M.K.S. (2020). Results of a 2-Year Prospective Multicenter Study Evaluating Long-term Audiological and Clinical Outcomes of a Transcutaneous Implant for Bone Conduction Hearing. Otol. Neurotol..

[20] (2019). Food and Drug Administration (FDA). https://www.accessdata.fda.gov/cdrh_docs/pdf19/K191921.pdf.

[21] Food and Drug Administration (FDA) (2003). Implantable Middle Ear Hearing Device—Guidance for Industry and FDA Staff.

[22] Drummond M. (2001). Introducing economic and quality of life measurements into clinical studies. Ann. Med..

[23] Wang S., Gum D., Merlin T. (2018). Comparing the ICERs in Medicine Reimbursement Submissions to NICE and PBAC-Does the Presence of an Explicit Threshold Affect the ICER Proposed?. Value Health.

[24] Tacconelli E. (2010). CRD’s Guidance for Undertaking Reviews in Health Care. Lancet Infect. Dis..

[25] Crowson M.G., Tucci D.L. (2016). Mini Review of the Cost-Effectiveness of Unilateral Osseointegrated Implants in Adults: Possibly Cost-Effective for the Correct Indication. Audiol. Neurotol..

[26] Lee C., Yeung M., Falk L., Ali A., Walter M. (2020). Implantable Devices for Single-Sided Deafness and Conductive or Mixed Hearing Loss: A Health Technology Assessment. Ont. Health Technol. Assess. Ser..

[27] Amin N., Soulby A.J., Borsetto D., Pai I. (2021). Longitudinal economic analysis of Bonebridge 601 versus percutaneous bone-anchored hearing devices over a 5-year follow-up period. Clin. Otolaryngol..

